# Research progress on pleiotropic neuroprotective drugs for traumatic brain injury

**DOI:** 10.3389/fphar.2023.1185533

**Published:** 2023-07-05

**Authors:** Qinghui Zhao, Huige Li, Hongru Li, Jianhua Zhang

**Affiliations:** ^1^ Institute of Physical Culture, Huanghuai University, Zhumadian, China; ^2^ Zhumadian Central Hospital, Zhumadian, China

**Keywords:** traumatic brain injury, neuroinflammation, therapeutic drugs, hydrogen, effect of treatment

## Abstract

Traumatic brain injury (TBI) has become one of the most important causes of death and disability worldwide. A series of neuroinflammatory responses induced after TBI are key factors for persistent neuronal damage, but at the same time, such inflammatory responses can also promote debris removal and tissue repair after TBI. The concept of pleiotropic neuroprotection delves beyond the single-target treatment approach, considering the multifaceted impacts following TBI. This notion embarks deeper into the research-oriented treatment paradigm, focusing on multi-target interventions that inhibit post-TBI neuroinflammation with enhanced therapeutic efficacy. With an enriched comprehension of TBI’s physiological mechanisms, this review dissects the advancements in developing pleiotropic neuroprotective pharmaceuticals to mitigate TBI. The aim is to provide insights that may contribute to the early clinical management of the condition.

## 1 Introduction

Traumatic brain injury (TBI) is the destruction or dysfunction of brain tissue structure caused by blunt mechanical external force on the head. Frequent traffic accidents, construction accidents, and violent injuries in modern society are important reasons for the increasing incidence of TBI yearly. Clinical manifestations of TBI patients extend from minor alterations in consciousness to enduring comatose states, with symptoms of swelling often accompanying diffuse brain damage effects. Globally, about 70 million people suffer a traumatic brain injury each year, which can have serious physical, psychosocial and economic consequences for patients, their families and society (Wiles, 2022). TBI is a serious public health problem, costing at least $400 billion annually (Maas et al., 2022). Even though the overall TBI mortality rate has decreased from 50% to 30% in the past decade. Nonetheless, 10% of patients suffering from mild TBI will still endure permanent neurological impairment, and between 66% and 100% of those with moderate to severe TBI will experience temporary or permanent disability or even death. These outcomes result in a significant societal and familial burden (Jiang et al., 2019).

Clinically, brain injury after traumatic brain injury generally includes two aspects: On the one hand, direct injury at the site of trauma, also known as primary brain injury, the clinical severity of primary brain injury depends on the site, nature and degree of injury, as well as the patient’s age, gender, previous medical condition, drug use history and alcohol use history. It can be treated by simple surgical operation in clinic. On the other hand, secondary injury is mediated by pathological processes such as post-traumatic ischemia and hypoxia, abnormal calcium channels, lipid peroxidation, neuroinflammation, excitatory amino acid toxicity, mitochondrial dysfunction, oxidative stress, calcium overload, and blood-brain barrier disruption (Jassam et al., 2017; Meshkini et al., 2017). The focus of clinical treatment after TBI is mainly to protect nerve damage after secondary brain injury and control the development of neuroinflammation, but there is currently no drug that can completely repair damaged nerves. Notably, neuroinflammation can exacerbate nerve cell damage and impede nerve repair, but it can also potentially promote tissue repair to some extent. Neuroinflammation, a relatively enduring mechanism of secondary cell death currently identified, provides a therapeutic window to control progressive brain tissue damage and enhance nerve function (Brett et al., 2022). This article primarily consolidates and elaborates on the research progress of pleiotropic neuroprotective drugs post-TBI.

## 2 TBI and neuroinflammation

TBI inflicts primary brain damage, prominently characterized by the disruption of the cellular membrane, blood vessels, and blood-brain barrier due to mechanical injury. Secondary brain damage, however, intensifies neurological impairment based on the foundation of primary injury through several mechanisms. These include glutamate excitotoxicity, intracellular calcium ion imbalance, free radical formation and amplified lipid peroxidation, mitochondrial dysfunction, inflammation, apoptosis, and diffuse axonal injury (Manivannan et al., 2021; Brett et al., 2022; Kalra et al., 2022). For instance, glutamate excitotoxicity is the overstimulation of glutamate excitability via the rise in extracellular glutamate levels and/or alterations in ionic glutamate receptors, culminating in cell toxicity. Mitochondrial dysfunction primarily obstructs cellular energy supply and oxidation, while inflammation induces severe brain parenchymal damage by releasing inflammatory factors (Manivannan et al., 2021). Moreover, it is established that various mechanisms of secondary brain injury post-TBI are interrelated and reciprocally influencing. Secondary brain injury serves as the pathological basis for death and disability in TBI patients.

Neuroinflammation following TBI primarily manifests in the following ways (Kalra et al., 2022): (i) Inflammation results in the breakdown of the blood-brain barrier, leading to the release of macrophages, neutrophils, and lymphocytes at the injury site. Ma et al. (2020) study found significant infiltration and aggregation of immune cells in the brain parenchyma, as evidenced by the co-localization of the macrophage marker CD68 and the tight junction protein ZO-1. (ii) When microglia overreact or activate, the subsequent release of oxidative metabolites and proinflammatory cytokines has detrimental effects on neurons. Inflammation promotes the overexpression of inflammatory factors in brain tissue, resulting in neuronal degeneration and death. TBI can trigger astrocytes to produce necrosis factor-α (TNF-α), interleukin-6 (IL-6), apolipoprotein E (ApoE), α1-antichymotrypsin (α1-ACT), α2-macroglobulin (α2 MAC), c-reactive protein (CRP), and extracellular enzymes such as the S100β protein, which contribute to neurotoxicity (Meshkini et al., 2021).

Currently, drugs aimed at the inflammatory response fall into three categories: the first seeks to manage the acute proinflammatory response at a level necessary to clear debris and danger signals; the second aims to stimulate the immunophenotype of anti-inflammatory and regenerative cells; and the third aims to reinstate normal function by timely prevention of chronic neuroinflammation development. Cell-based therapies can potentially mitigate neuroinflammation and enhance functional recovery following TBI (Xiong et al., 2018).

## 3 TBI and pleiotropic neuroprotection

Neuroprotection typically refers to preserving the neuronal structure and sustaining neural function. Pleiotropic neuroprotection represents an evolution of the traditional concept of neuroprotection, emphasizing a therapeutic approach that simultaneously blocks multiple targets, superseding the single-target treatment paradigm. The goal is to provide neuroprotective effects for various forms of brain injury, including TBI, intending to achieve more optimal therapeutic outcomes (Scarboro and McQuillan, 2021). Historically, research on therapeutics for TBI treatment has yielded minimal effects due to the focus on a singular target and misconceptions in assessing clinical symptoms of TBI. The Glasgow Coma Scale (GCS) is commonly utilized to assess TBI severity in post-TBI patients, categorizing them into mild (GCS score 13-15), moderate (GCS score 9-12), or severe (GCS score 3-8) (Di Pietro et al., 2020). However, it is noted that neuronal damage is ubiquitous in both mild and severe TBI, and less than 40% of moderate to severe TBI patients previously employed can return to their jobs and achieve a normal neurocognitive status one-year post-discharge from the hospital (Hart et al., 2019).

**FIGURE1:**
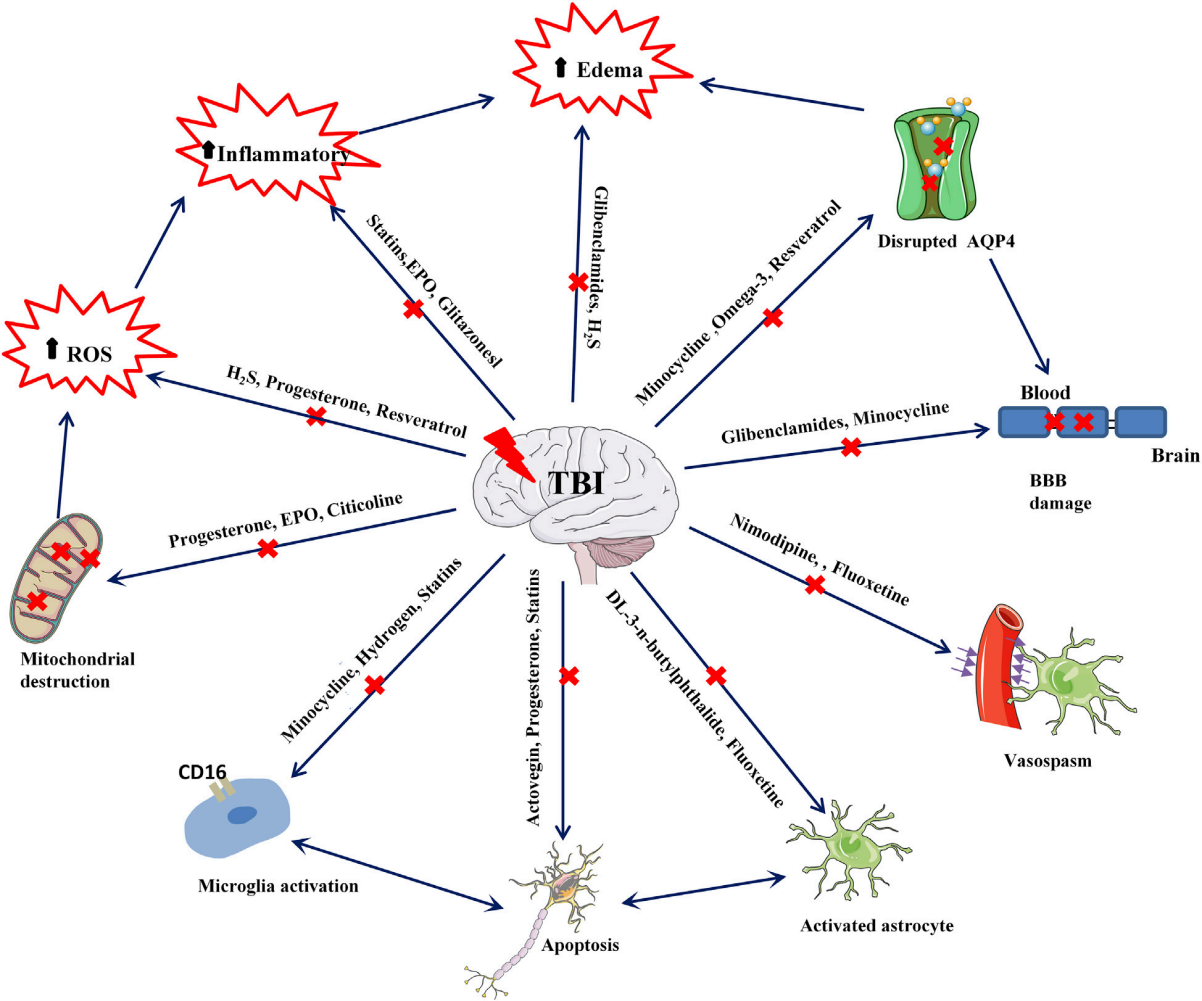
Summary of targets for drug therapy after TBI.

Recent neuronal studies suggest that neuroprotection should extend beyond neurons due to the complex interplay between glial and endothelial cells. The focus has expanded to the preservation of glial cells (including astrocytes, microglia, and oligodendrocytes) and vascular cells (including endothelial cells, smooth muscle cells, and pericytes) to bolster neuroprotection (Lerouet et al., 2021). In the past decade, preclinical researchers have extensively studied pleiotropic neuroprotection post-TBI, and many drug molecules have shown promising results in animal models. However, it is unfortunate that these molecules have yet to demonstrate effective neuroprotective activity in clinical stages. Several possible explanations have been proposed for this (Wei and Xiao, 2013; Duncan, 2020; Dams-O'Connor et al., 2023; Kundu and Singh, 2023): (i) Established animal models are restrictive, often unable to account for the precision required in model creation and myriad factors, including the location and severity of injuries in actual TBI patients. Hence, the efficacy of preclinical neuroprotection drugs remains limited to animal models. (ii) Pharmacokinetic and pharmacodynamic modeling studies of drugs are insufficiently detailed. In preclinical studies, drugs are typically administered immediately following TBI model establishment, whereas, in actual clinical practice, it is challenging to initiate treatment at the earliest stage following TBI. Furthermore, there is a paucity of useful data on drug plasma protein binding times post-treatment, effective drug concentrations, and the influence of metabolite activity on treatment. (iii) Differences in dosage and administration methods exist due to preclinical trials being conducted on rats, mice, or other animal models, resulting in substantial disparities in anatomy, metabolism, and neurobiology between animals and humans. Furthermore, in clinical trial stages, considerations of drug toxicity and side effects often lead to less frequent and smaller dosages. (iv) There is a lack of awareness concerning pleiotropic neuroprotection. When developing drugs, many preclinical researchers concentrate on a single target molecule, making it difficult to contemplate the interactions among multiple targets. Secondary brain injury in TBI patients is a remarkably complex process, and various targets interact with each other. Therefore, the simultaneous targeting of multiple facets by pleiotropic drugs should be contemplated for effective nervous system protection (Figure 1).

## 4 TBI and pleiotropic neuroprotective drugs

### 4.1 Progesterone

Progesterone, as a steroid hormone, is generally secreted by the ovary and placenta, but in the nervous system, astrocytes, oligodendrocytes, and microglia can also secrete progesterone. Progesterone Receptor mediates the regulatory effect of progesterone on the nervous system. Many animal experimental models have confirmed that progesterone can effectively reduce cerebral vasodilatation, protect and restore the brain barrier, improve muscle survival, and limit cell necrosis and apoptosis, playing a role in the pleiotropic neuroprotection of patients after TBI (Nasre-Nasser et al., 2022). In 2020, Sara et al. (2020) conducted a study using the nerve growth factor (NGF)/interleukin-6 (IL-6) ratio to evaluate the neuroprotective effect of progesterone on experimental diffuse brain injury. They found that in rats treated with progesterone a week after injury, there was a significant decrease in brain edema and NGF and IL-6 levels in serum and cerebrospinal fluid. Furthermore, the NGF/IL-6 ratio in the cerebrospinal fluid of the treated rats increased significantly, suggesting that progesterone might exert its neuroprotective role by enhancing NGF and decreasing IL-6.

Moreover, results from a study using a rat model of transient middle cerebral artery occlusion revealed that progesterone treatment reduced not only mitochondrial reactive oxygen species (ROS) production and inhibited mitochondrial permeability transition pore (mPTP) opening but also improved mitochondrial membrane potential and respiratory rate. These findings indicate that progesterone positively influences mitochondrial biological function, delivering a favorable neuroprotective effect (Andrabi et al., 2017).

Paclitaxel (Taxol), a natural bioactive compound with progesterone-like properties, has been shown to inhibit both microglial activation and oxidative tissue damage, thus providing a pleiotropic neuroprotective effect against cell apoptosis. The underlying mechanisms were demonstrated to be as follows (Inoue et al., 2019): (i) Paclitaxel enhances the expression level of vascular endothelial growth factor-D (VEGF-D), thereby protecting central neuronal function and improving cognitive abilities. (ii) Paclitaxel effectively inhibits neuroinflammatory response by suppressing the expression of the triggering receptor 2 (TREM2) on bone marrow cells expressed by microglia. (iii) Paclitaxel reduces nitrite synthesis by inhibiting the expression of inducible nitric oxide synthase (iNOS) and cyclooxygenase-2 (COX-2) in microglia, consequently offering neuroprotective effects.

Despite numerous animal studies demonstrating the significant neuroprotective effect of progesterone on TBI, Phase III clinical trials have not been able to show a clinical benefit of progesterone therapy (Skolnick et al., 2014; Wright et al., 2014; Stein, 2015). This might be related to varying patient characteristics, injury locations, and severity.

### 4.2 Statins

Statins, known for inhibiting 3-hydroxy-3-methylglutaryl coenzyme A (HMG-CoA) reductase, are crucial for their lipid-lowering and cholesterol-lowering effects in clinical applications. However, recent studies have suggested that statins may also confer neuroprotective effects in several neurological conditions, including moderate brain injury, stroke, subarachnoid hemorrhage, and non-traumatic cerebral hemorrhage (Robertson et al., 2017; Susanto et al., 2023). Additional research has demonstrated that statins like simvastatin can inhibit cholesterol biosynthesis, prevent caspase-3 activation, and apoptotic cell death, thereby fostering functional recovery and neuronal salvage after TBI. Furthermore, simvastatin boosts the levels of several growth factors and stimulates neurogenesis (Crupi et al., 2020).

In another study using a rat model of fluid percussion brain injury, atorvastatin (1 mg/kg per day for 2 weeks; Pfizer Pharmaceutical Co., LTD. H20051408) was shown to inhibit the expression levels of IL-6, TNF-α, and IL-1β in serum. It also decreased the injury of brain tissue markers such as Toll-like receptor 4 (TLR4), nuclear factor kappa-light-chain-enhancer of activated B cells (NF-kBp65), a phosphorylated inhibitor of kappa B (p-IkB), and cleaved caspase-3. These findings indicate that atorvastatin might inhibit the release of inflammatory factors and neuronal apoptosis by regulating the TLR4/NF-kB signaling pathway in TBI rats (Bagheri et al., 2020; Lokhandwala et al., 2020). A randomized, double-blind, placebo-controlled clinical trial enrolling 65 patients with TBI showed that although atorvastatin was not more effective than placebo in reducing the rate of contusion expansion, the atorvastatin group showed significantly better scores in the modified Rankin Scale (MRS), Glasgow Outcome Scale (GOS), and Disability Rating Scale (DRS) functional outcome scale 3 months after moderate to severe TBI (Farzanegan et al., 2017). These promising results suggest the need for larger, multi-center clinical trials to validate these findings.

### 4.3 Erythropoietin and its derivatives

Erythropoietin (EPO) is a glycoprotein hormone primarily known for promoting the differentiation and maturation of erythroid progenitor cells, thereby boosting erythropoiesis and ultimately enhancing oxygen-carrying capacity. In addition to these roles, EPO has demonstrated anti-inflammatory, anti-apoptotic, anti-oxidative, and angiogenic properties, along with cytoprotective effects on endothelial cells, neurons, and glial cells (Katiyar et al., 2020). Due to its extensive biological activities, numerous studies have investigated the potential neuroprotective effects of EPO. Among these, a study found that EPO can prevent the apoptosis of nerve cells induced by factors such as calcium overload, membrane peroxidation, and free radical damage. In this study, rats were administered 1,000 U/kg EPO intraperitoneally 30 min after TBI, resulting in reduced brain nerve function damage caused by inflammation and positive effects on injury repair and nerve function recovery (Tunc Ata et al., 2016). The researchers injected rhEPO 5,000 IU/kg intraperitoneally every day for 7 days post-TBI, leading to significant increases in the volume density, specific surface area, specific membrane area, and number density of mitochondria in rat brain tissue. This implies that EPO can inhibit neuroinflammation and restore the function of damaged mitochondria to some extent. In addition to this, recent studies found that rhEPO can inhibit mitochondrial damage and improve redox imbalance and neuroinflammation in 1-methyl-4-phenyl-1,2,3,6-tetrahydropyridine (MTPT) stimulated mice by maintaining redox balance and acting on both mitochondrial and glycolytic process levels. This ultimately inhibits the degeneration of dopaminergic neurons, restores their cell vitality, and achieves a protective effect on nerves (Zhu et al., 2009; Rey et al., 2021). However, the results of a double-blind, randomized controlled trial in patients with TBI by Robertson et al. (2014) showed that EPO administration did not improve neurological function in patients with closed craniocerebral injury. Nichol et al. (2015) conducted a double-blind, placebo-controlled trial (EPO-TBI) at 29 centers (university-affiliated teaching hospitals) in seven countries (Australia, New Zealand, France, Germany, Finland, Ireland, and Saudi Arabia). The effects of EPO on neurological recovery, mortality, and venous thrombotic events in patients with traumatic brain injury were investigated. The results showed that EPO did not reduce the number of patients with severe neurological dysfunction (GOS-E levels 1-4) or increase the incidence of deep venous thrombosis in the lower extremities after moderate-severe craniocerebral trauma. The effect of EPO on mortality from moderate to severe craniocerebral injury remains uncertain.

### 4.4 Hydrogen

Hydrogen is the lightest known gas, and due to its small molecular size, it can easily cross the blood-brain barrier, providing neuroprotection (Li et al., 2020; Wang et al., 2020). Research suggests that hydrogen may be beneficial in various neurological conditions such as spontaneous hypertensive cerebral hemorrhage, hypoxic-ischemic encephalopathy, brain trauma, cerebral infarction, and subarachnoid hemorrhage, as it has anti-inflammatory, anti-oxidative, and anti-apoptotic properties. In a study using controlled cortical impact injury model rats, hydrogen inhalation was applied for 1 hour immediately following TBI with a hydrogen concentration set at 4%. The results showed a significant decrease in proinflammatory cytokines IL-1β, IL-17, IL-6, IL-13, interferon-γ (IFN-γ), IL-2, and IL-5 in the serum at 2 h post-TBI, demonstrating an inhibitory effect of hydrogen on acute inflammatory response post-TBI. There was also an increase in macrophage colony-stimulating factor (M-CSF), growth-associated oncogene (GRO-KC), granulocyte colony-stimulating factor (G-CSF), macrophage inflammatory protein-3α (MIP-3α), IL-7, IL-12, and IL-1α, suggesting a protective effect through the upregulation of chemokines for neural recovery (Zhao et al., 2020).

In another study, high-concentration hydrogen inhalation (42%) was applied to rats, and after 2 days, the modified Neurological Severity Score (mNSS) was significantly decreased. There was an upregulation of myeloperoxidase (MPO) and HO-1 expression, downregulation of the apoptosis-related proteins Bax and caspase-3, and upregulation of Bcl-2 expression. These results suggest that oxidative stress and apoptosis around the traumatic area of the cerebral cortex are significantly reduced, and neurological function scores of rats after brain trauma are significantly improved, potentially related to the anti-oxidative stress and anti-apoptosis response of hydrogen. Additionally, Fu et al. (2018) studied the effects of hydrogen-rich water on inflammatory factors and mitochondrial damage in rats with TBI using intraperitoneal injection of hydrogen-rich water (5 mL/kg). The results showed that hydrogen-rich saline could significantly inhibit the expression of Bax protein and promote the expression of Bcl-2 protein. Also, according to the reduction of mitochondrial ROS and the increase of mitochondrial membrane potential (MMP) in brain cells of TBI rats treated with hydrogen-rich saline, the mPTP is decreased, suggesting that hydrogen-rich saline can reduce mitochondrial dysfunction of brain cells after TBI, thereby reducing nerve cell apoptosis. Hydrogen can also affect energy and nucleotide metabolism after TBI and alter pathological gene expression related to oxidation, carbohydrate metabolism, and inhibition of cytokine activation (Hu et al., 2021).

However, while there has been considerable study of the protective effects of hydrogen in TBI, most of the research is based on animal and cell models. Currently, no relevant clinical studies have been reported. Clinical studies need to confirm the safety and efficacy of hydrogen intervention in treating TBI. High-quality clinical trials, such as multi-center randomized controlled, double-blind trials, are essential to advancing the use of hydrogen in the clinical treatment of TBI.

### 4.5 Resveratrol

Resveratrol (Res) is a polyphenolic compound derived from plants with a stilbene structure. It has demonstrated beneficial pharmacological activity in anti-tumor, antibacterial, and cardiovascular system protection. It primarily treats obesity, diabetes, and cardiovascular diseases in China (Galiniak et al., 2019; Meng et al., 2021). Recent studies have indicated that Res has significant neuroprotective effects in TBI models (Martin et al., 2015). Additionally, dietary polyphenol resveratrol (RV) displays noticeable antioxidant and cellular protective effects, exerting its biological activity through various molecular pathways and ultimately providing neuroprotective effects across diverse organisms (Griñán-Ferré et al., 2021). In 2018, Zou et al. (2018) used a free-fall device to create a TBI rat model and studied the inhibition of sirtuin 1 (SIRT1) and activation of inflammasome 3 (NLRP3) to alleviate traumatic brain injury in rats. Using neuron-specific enolase (NSE) and brain water content (BWC), specific biomarkers closely associated with neuronal damage, to indicate the extent of brain damage, they found that Res treatment significantly reduced the elevation of NSE and BWC. Res also reduced the activation of NLRP3, caspase-1, and the release of IL-1β and IL-18. These results suggest that Res may protect TBI by inhibiting the NLRP3 inflammasome signaling pathway. The same study found that when rats pretreated with Res also received sirtinol, the blockade of SIRT1 activation by sirtinol was associated with enhanced NLRP3 inflammasome activation in TBI. This suggests that the mechanism by which Res inhibits NLRP3 inflammasome is SIRT1-dependent. Other studies (Peng et al., 2022) have confirmed that Res can also inhibit inflammation and apoptosis through the SIRT1/NF-κβ pathway, thereby protecting the nervous system.

Pterostilbene (PTE), a methylated derivative of Res, is widely distributed in various plants and used clinically for anti-tumor, anti-oxidative, anti-inflammatory, and neuroprotective effects (Liu et al., 2020). *In vitro* cell studies of PTE’s neuroprotective mechanisms found that PTE treatment significantly inhibited the expression of Bdnf and Bcl-2 miRNA and upregulated the expression of miR-702-5p in H19-7 cells. This suggests that PTE protects the nervous system by enhancing cell viability and inhibiting cell apoptosis (Weisz et al., 2022). PTE’s inhibitory mechanism on oxidative stress primarily involves the following: (i) PTE increases the expression level of manganese superoxide dismutase (Mn-SOD) in the mitochondrial matrix by activating Bcl-2 associated X protein, thereby inhibiting mitochondrial respiration and reducing oxide production. (ii) PTE reduces the production of reactive oxygen species (ROS), which are essential for oxidative stress, by inhibiting low-density lipoprotein receptor-1 (LOX-1). (iii) PTE can activate nuclear-factor-E2-related factor2 (Nrf2) and increase the expression of Nrf2 downstream target genes such as heme oxygenase-1, carnitine acetyltransferase, superoxide dismutase (SOD), and glutathione peroxidase to achieve antioxidant effects. A substantial amount of preclinical data suggests that Res can be used in clinical trials to treat TBI.

### 4.6 Minocycline

Minocycline (MINO) is a broad-spectrum antibiotic belonging to the tetracycline family, known for its lipophilic properties that allow it to cross the blood-brain barrier readily. This makes it capable of exerting anti-inflammatory, anti-apoptotic, and anti-oxidative effects. MINO is commonly used in the clinic to treat conditions like periodontitis and acne, but it is also considered an ideal candidate. Studies have shown that MINO, when administered 24 h post-TBI in a mouse model, can reduce the acute loss of neurons in the CA3 region of the hippocampus. This neuroprotective effect was verified by both flow cytometry 7 days after the event and by semiautomated quantitative morphometric measurements of hippocampal microglia (Celorrio et al., 2022). Further research established a TBI model in male mice using a controlled cortical impact (CCI) device, followed by an intraperitoneal injection of 45 mg/kg MINO 30 min post-TBI. The observed reduction in albumin and MMP-9 expression levels indicated that MINO could partially restore tight junction protein levels, thereby reducing the blood-brain barrier dysfunction following TBI. This helps maintain the integrity of the blood-brain barrier by inhibiting the expression of AQP4, effectively ensuring the recovery of neurological function (Lu et al., 2022).

MINO has also shown promise in dealing with nerve damage induced by a rat TBI model (Taylor et al., 2018; Zhang J. et al., 2020). Preclinical studies have demonstrated the neuroprotective effects of MINO, both when used alone and in combination with N-acetylcysteine (NAC) and N-acetylcysteine amide (NACA). MINO combined with NAC can improve memory and cognition in rats and repair white matter damage by protecting oligodendrocytes (Haber et al., 2018). Since oligodendrocyte loss can be observed post-TBI, the administration of MINO combined with NAC 12 h post-TBI has been seen to protect against oligodendrocyte apoptosis within a 2-14 days window (Sangobowale et al., 2018). Despite these promising results, MINO’s application for TBI treatment in humans needs more research. While a phase 1 clinical trial indicated that MINO was safe for TBI, a study involving 15 patients more than 6 months post-moderate/severe TBI showed that chronic microglial activation was suppressed with 100 mg of MINO administered twice daily. Although this may promote neuro repair, it can also lead to increased neurodegeneration, as indicated by raised plasma neurofilament light chain levels (Scott et al., 2018; Meythaler et al., 2019). Therefore, while MINO alone might not be effective in treating TBI, further human trials with MINO and NAC in combination may be warranted.

### 4.7 Protein-free calf blood extract

Deproteinated extract from calf blood (DECB) is a complex substance derived from the venous blood of young calves. This mixture primarily contains small molecular peptides, amino acids, adenosine, glycolipids, intermediate products of lipid metabolism, and a variety of inorganic ions. The primary mechanism of DECB is thought to be enhancement of the cellular glucose and oxygen uptake, improvement in cellular metabolism, promotion of glucose transport, stimulation of pyruvate dehydrogenase and glucose oxidation, and increase in the absorption and utilization of oxygen. It is used clinically to treat diseases caused by diabetes, brain disorders, mucosal injury, and ocular surface injury (Machicao et al., 2012; Firan et al., 2020). Another product derived from calf blood, Actovegin, is extensively used in the clinic for peripheral and cerebral circulation disorders, burns, impaired wound healing, radiation-induced injuries, and diabetic polyneuropathy. Preclinical studies have confirmed that Actovegin can protect multipotent nerves post-TBI by activating anti-oxidative responses and inhibiting apoptosis (Daia et al., 2021). Actovegin’s neuroprotective mechanisms are as follows: (i) It enhances oxygen and glucose uptake in the central nervous system, which increases energy metabolism and maintains glucose homeostasis in the CNS, thus protecting neurons and cognitive function. (ii) For Alzheimer’s disease characterized by the presence of amyloid β-polypeptide (Aβs), Actovegin can effectively mediate the inflammatory pathway caused by Aβs by increasing oxidative activation, which can improve cell apoptosis and protect the nervous system. (iii) Actovegin can inhibit the release of inflammatory factors by activating the NF-kB pathway, thereby achieving neuroprotection. Researchers have found that Actovegin can benefit TBI patients through clinical trials (Daia et al., 2021). However, the current research data of actovegin on TBI is still insufficient, and a large number of preclinical and clinical trials are still needed to verify the effect of DECB and actovegin on different types of TBI.

### 4.8 Hydrogen sulfide gas

Hydrogen sulfide (H_2_S), a toxic and pungent gas, is primarily used to treat cardiovascular diseases, neurological diseases, mental disorders, metabolic syndrome, and diabetes. Research suggests that neurons, microglia, and astrocytes produce H_2_S in the nervous system and has anti-oxidative, anti-inflammatory, and anti-apoptosis effects in the neuronal system, which could potentially help protect against secondary brain injury (Zhang L. et al., 2020). In addition, H_2_S can specifically interact with adenosine triphosphate-sensitive potassium channels to reduce neuronal excitability and utilize cystathionine-γ-lyase (CSE), a specific element in the central nervous system. A 2019 study by Shan Haiyan et al. explored the neuroprotective effect of exogenous H_2_S on brain injury by regulating endogenous H_2_S metabolism in mice. They found that pretreatment with sodium hydrosulfide (NaHS) significantly reduced intracerebral hemorrhage (ICH) content in mice, inhibited the upregulation levels of LC3 and Beclin-1, and improved motor and cognitive functions (Shan et al., 2019). Their study also found that the use of H_2_S in the rat model could protect hippocampal cells by inhibiting the occurrence of central nervous system stress responses. Furthermore, it could also reduce the release of inflammatory factors and control cell apoptosis by inhibiting the expression levels of p65 and pIkBα/IkBα. These results suggest that H_2_S has a multifaceted neuroprotective mechanism on the brain injury in rats. Studies on APP/PSI double transgenic mice have also demonstrated that an appropriate concentration of H_2_S could reduce Aβ production and improve mitochondrial function in APP/PSI neurons by selectively inhibiting the activity of γ-secretase (Zhao et al., 2016). Recent studies have shown significant improvements in brain lesion volume, brain edema, blood-brain barrier function, motor behavior function, and spatial memory energy in the H_2_S group compared to the model group after TBI (Jiang et al., 2013; Karimi et al., 2017). In a mouse TBI model, H_2_S improved the proliferation of apoptotic and autophagy markers and edema and cognitive deficits (Zhang et al., 2014). Despite the promising neuroprotective and restorative effects of H_2_S on the nervous system, providing a potential new avenue for TBI treatment, several questions remain. The precise mechanisms of H_2_S in the brain are still unclear. Furthermore, it is uncertain whether the concentration of H_2_S used in studies would harm humans and whether conclusions drawn from different models and reagents can be uniform. Before clinical trials can be conducted, the effects of H_2_S on TBI require further preclinical investigation.

### 4.9 Glitazones

Glitazones, known as thiazolidinediones, are synthetic peroxisome proliferator-activated receptor (PPAR) γ agonists primarily used to treat hyperglycemia and diabetes. PPAR, a nuclear receptor family member, regulates gene transcription during metabolic processes and cell differentiation. Three PPAR isotypes have been identified in mammals: PPARα (NR1C1), β/δ (NR1C2), and γ (NR1C3). All subtypes are expressed at different levels in the central nervous system (Cai et al., 2018). PPARγ is primarily expressed in neurons and astrocytes under physiological conditions (Ferguson et al., 2014; Warden et al., 2016), and its expression in microglia is increased in inflammatory states (Lerouet et al., 2021; Pinna, 2023). Recent studies have found that glitazones can reduce neuroinflammation and neuronal damage following brain injury (Sauerbeck et al., 2011; Liu et al., 2017; Deng et al., 2019). After an injury, a single dose of pioglitazone can even reduce cortical oxidative damage and the microglial response (Pilipović et al., 2015). Das et al. (2019) used a rat model of traumatic brain injury and found that pioglitazone treatment post-injury could activate PPARγ, reduce CCL20 and IL-1β, and decrease the neuroinflammatory response, thereby improving neuronal function. Ratliff et al. (2020) applied pioglitazone after TBI in a repetitive mild TBI model. They observed significant morphological improvements in the dendrites and dendritic spines of neurons in the dentate gyrus of the hippocampus, ultimately ameliorating symptoms such as memory deficits. In another study, Yao et al. (2015) established a rat CCI brain injury model and administered rosiglitazone intraperitoneally post-injury. They found that rosiglitazone could reduce the expression levels of tumor necrosis factor α and interleukin-6 proteins after brain injury, significantly decreasing neuronal apoptosis and autophagy and promoting functional recovery. The abundance of preclinical data suggests that glitazones may be promising for clinical trials aimed at treating TBI.

### 4.10 Glibenclamides

Glibenclamide is a drug widely used in type 2 diabetes management, acting as an antagonist of the Sulfonylurea receptor 1–transient receptor potential melastatin 4 (SUR1-TRPM4). This unique ion channel, discovered via single-channel patch-clamp experiments, is not expressed in normal brain tissue. However, following brain injury, SUR1-TRPM4 expression is upregulated in various central nervous system cell types, including neurons, microglia, astrocytes, and endothelial cells (Patel et al., 2010; Martínez-Valverde et al., 2015; Gerzanich et al., 2019). The SUR1-TRPM4 channel has been associated with various acute and chronic central nervous system diseases in preclinical studies using rodent models. These include focal cerebral ischemia (Simard et al., 2006; Wali et al., 2012), global cerebral ischemia due to cardiac arrest (Huang et al., 2015; Huang et al., 2016; Huang et al., 2018; Nakayama et al., 2018), TBI (Zweckberger et al., 2014; Xu et al., 2017; Jha et al., 2018; Kochanek et al., 2018; Jha et al., 2020; Jha et al., 2021; Zusman et al., 2023), and spinal cord injury (Hosier et al., 2015). It is also related to subarachnoid space, intracerebral and intraventricular hemorrhage (Simard et al., 2009), cerebral edema due to metastatic tumors (Thompson et al., 2013), early-produced hemorrhagic encephalopathy (Tosun et al., 2013), neuropathic pain from peripheral nerve injury, experimental allergic encephalomyelitis (EAE), and EAE-associated optic neuritis (Schattling et al., 2012; Makar et al., 2015; Gerzanich et al., 2017).

Glibenclamide, as an antagonist of SUR1-TRPM4, has shown a protective effect across these conditions. In ischemic or TBIs, glibenclamide’s interaction with SUR1-TRPM4 reduces depolarization and lessens blood-brain barrier leakage and brain edema formation. This occurs both in rodent models and humans, although the full complexity of the underlying mechanism is yet to be fully understood (Pergakis et al., 2019). A Phase II clinical trial is underway to determine whether glibenclamide can reduce posttraumatic edema and/or bleeding compared to a placebo (Pergakis et al., 2019; Lerouet et al., 2021). The results of this trial could further elucidate the role of glibenclamide in managing brain injuries.

### 4.11 Amantadine

Amantadine is a dopaminergic drug that is an antagonist of N-methyl-D-aspartate (NMDA) and can increase the release of dopamine through presynaptic action or inhibition of dopamine reuptake; It also alters the structure of dopamine receptors through postsynaptic action. It can prevent glutamate-induced excitatory toxicity by acting non-competitively on NMDA receptor antagonists (Tan et al., 2015). The FDA has approved it to prevent influenza and Parkinson’s disease (Rascol et al., 2021). Amantadine is most commonly used in patients with consciousness disorders and those undergoing inpatient neurological rehabilitation, although the mechanism of action is unknown. However, several clinical trials have shown a positive effect of amantadine in neurobehavioral recovery, cognitive function, and improved disability score after TBI (Loggini et al., 2020; Ma et al., 2020; Ma and Zafonte, 2020; Ahmed, 2022). Clinical studies have shown that amantadine is well tolerated and can accelerate the rate of cognitive recovery during the middle stages of TBI. However, in a 2018 study, a multi-site randomized controlled trial did not support using amantadine to improve cognitive performance after TBI (Hammond et al., 2018). The long-term role of amantadine in cognitive recovery is not well defined, and further large randomized clinical trials in refined subgroups of patients are needed to define its use better. Amantadine is currently undergoing Phase IV clinical trials.

## 5 Summary

TBI is a highly heterogeneous condition that reflects a variety of macroscopic modes of injury, such as extrinsic compression from the mass lesion, contusion, and diffuse axonal injury. It also comprises a range of mechanisms leading to neuronal injury, including “classical” ischemia, apoptosis, mitochondrial dysfunction, cortical spreading depression, and microvascular thrombosis. The resultant clinical courses vary extensively (Khellaf et al., 2019). Over the past three decades, many preclinical experiments have been conducted to develop hundreds of neuroprotective drugs for TBI treatment. Although these drugs show significant efficacy and minor toxic side effects in animal models, nearly 40 preclinical drugs have failed in phase II or III clinical trials (Alves et al., 2019). (Table1)

**TABLE 1:** Summary of pleiotropic neuroprotective drugs in TBI.

Drug	Mechanism	Summary of results	Status	Authors and year
Progesterone	Reduce ROS generation	Reduce cell apoptosis	Terminated at Phase III	Stein (2015)
	Reduces inflammation	Reduce mortality		
	Improve mitochondrial function	Improve patient awareness		
Statins	Reduces inflammation	Improved neuron repair	Phase I/II	Xiong et al. (2018)
	Inhibit caspase-3 activation	Reduce mortality rates		
	Decreased activation of microglia	Promote functional recovery		
Erythropoietin	Reduces inflammation	Reduce cell apoptosis	Phase III	Nichol et al. (2015)
	Improve mitochondrial function	Protect neurons		
	Inhibition of mitochondrial damage	Improve neurological recovery		
Hydrogen	Reduce ROS generation	Reduce cell apoptosis	Preclinical study	Zhao et al. (2020)
	Reduces inflammation	Protect neurons		
	Decreased activation of microglia	Improve neurological recovery		
Resveratrol	Anti-oxidative stress	Reduce cell apoptosis	Preclinical study	Feng et al. (2022)
	Inhibition of NLRP3 inflammasome	Decrease brain water content		
	Activation of Bcl-2 related proteins	Protect neurons		
Minocycline	Decreased expression of MMP-9	Reduce BBB dysfunction	Phase IIα	Meythaler et al. (2019)
	Restore the tight junction protein	Improves memory and cognition	Phase III	Koulaeinejad et al. (2019)
	Decreased activation of microglia	Improve nerve repair function		
Actovegin	Increase the cellular energy metabolism	Reduce cell apoptosis	Phase II	Daia et al. (2021)
	Increased oxygen and glucose intake	Protect neurons		
	Reduces inflammation	Improve cognitive function		
H_2_S	Anti-oxidative stress	Reduce cerebral edema	Preclinical study	Zhang et al. (2020a)
	Reduces inflammation	Improves motor function		
	Reduces the production of neurons Aβ	Improves cognitive function		
Glitazones	Activate PPARγ	Improved memory deficits	Preclinical study	Ratliff et al. (2020)
	Reduce CCL20 and IL-1β	Improve nerve function		
	Reduce neuroinflammatory response	Improves cognitive function		
Glibenclamides	SUR1-TRPM4 antagonist	Reduce BBB damage	Phase II	Pergakis et al. (2019)
		Reduce cerebral edema		
Amantadine	An antagonist of NMDA	Accelerate cognitive recovery	Phase IV	Hammond et al. (2018)
	Increases the release of dopamine	Improved behavior recovery		
		Improved disability score		

The neuroinflammatory response after TBI can cause multiple brain abnormalities, with increased levels of inflammatory biomarkers being a typical manifestation. This article primarily reviews neuroprotective drugs used post-TBI in recent years, including their molecular and cellular regulatory mechanisms. Although most drugs fail in clinical applications, it does not imply they lack efficacy. In fact, their failures can stimulate preclinical researchers to continue exploring drugs that may eventually be applied for pleiotropic neuroprotection post-TBI. Alternatively, these drugs may yield better results in treating other diseases.

In conclusion, decades of clinical translation failures have made us appreciate the need for preclinical research closer to clinical situations. This includes considering individual patient factors and conducting multiple drug trials on different trauma models. The ultimate goal is to reduce neuronal injury treatment for clinical TBI patients and improve patient prognosis.
